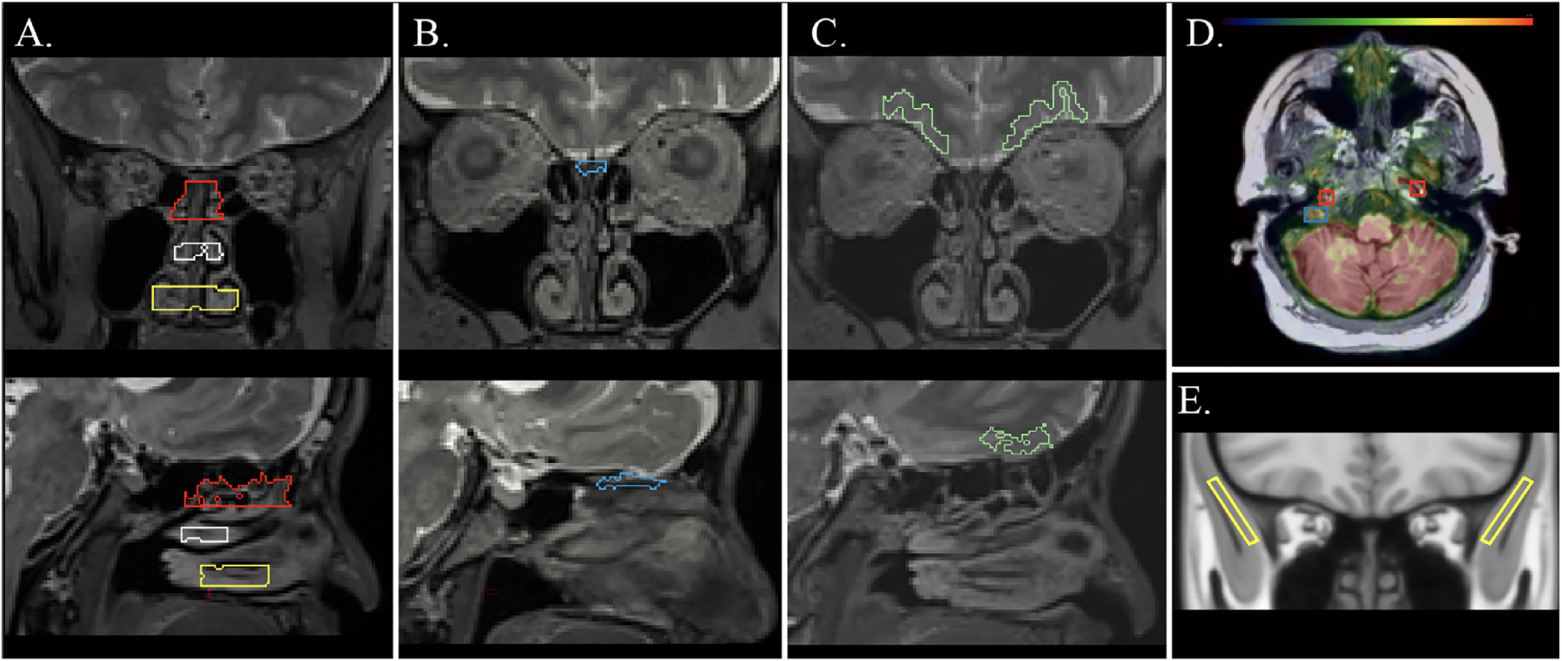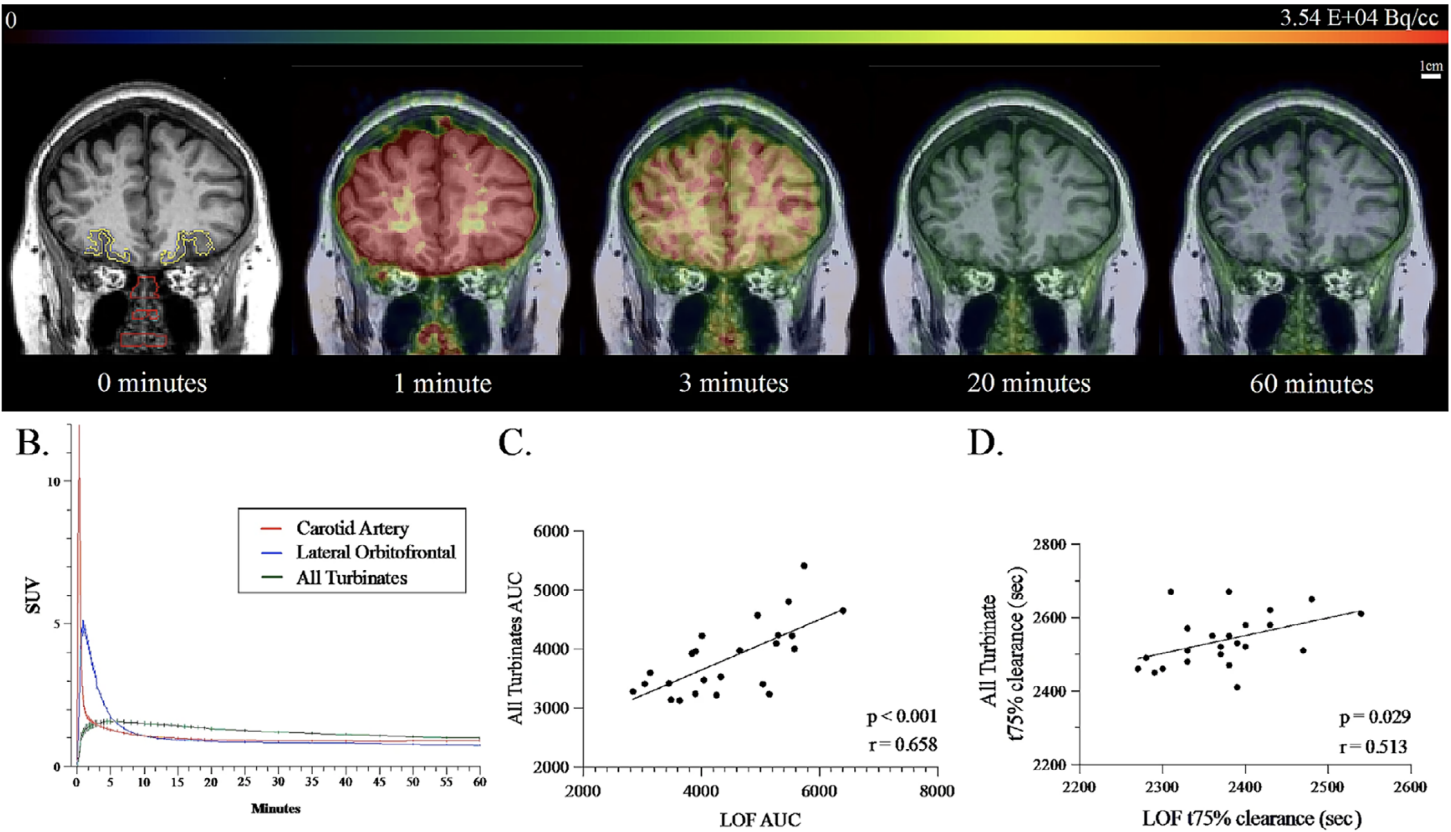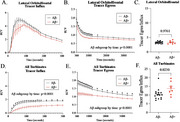# Impaired Brain to Nasal Turbinates Pathway in Aging Amyloid Positive Subjects Using [1‐11C]‐Butanol PET Imaging

**DOI:** 10.1002/alz70856_100912

**Published:** 2025-12-25

**Authors:** Samantha A Keil, Xiuyuan Hugh Wang, Neel H Mehta, Liangdong Zhou, Jana Ivanidze, Tracy A Butler, Kewei Chen, Henry Rusinek, Roxana O. O Carare, Yi Li, Gloria Chiang, Mony J. de Leon

**Affiliations:** ^1^ Weill Cornell Medicine, New York, NY, USA; ^2^ Weill Cornell Medicine, New York City, NY, USA; ^3^ Harvard, Cambridge, MA, USA; ^4^ NewYork Presbyterian, New York City, NY, USA; ^5^ Banner Alzheimer's Institute, Phoenix, AZ, USA; ^6^ New York University Grossman School of Medicine, New York, NY, USA; ^7^ University of Southampton, Southampton, Hampshire, United Kingdom

## Abstract

**Background:**

Reduced cerebrospinal fluid (CSF) clearance is a suggested pathological feature of Alzheimer's disease (AD). There is increasing evidence from non‐human studies that the nasal mucosa may serve as a CSF drainage site, particularly through the cribriform plate into the nose. We explored this pathway using dynamic PET with [1‐11C]‐Butanol, a radiotracer with high permeability and minimal brain binding, to examine the relationship between egress from the brain and the nasal turbinates, particularly in the context of amyloid pathology.

**Methods:**

Cognitively normal subjects (*n* = 24; 65+ years) underwent 60 minutes of dynamic PET [1‐11C]Butanol imaging. The cohort consisted of 8 amyloid PET Aβ+ and 16 amyloid PET Aβ‐ participants. Regions of interest (ROI) included the lateral orbitofrontal cortex (LOF), cribriform plate, and the superior, middle and inferior nasal turbinates. Time‐activity curves were analyzed to model tracer influx, egress, and area under the curve (AUC) to assess drainage kinetics.

**Results:**

Substantial positive correlations were observed between tracer kinetics in the LOF and turbinates (Spearman correlation r = 0.658, *p* < 0.001), indicating a functional connection between brain and nasal clearance pathways. In subjects with amyloid positivity, tracer input into and egress from the nasal turbinates were significantly reduced, as indicated by time‐activity curves (TACs) from [1‐11C]‐Butanol administration. More specifically, there was a significant main effect of Aβ subgroup (F(1,22) = 4.641, *p* =  0.0424) on egress from the LOF, as well as input into (F(1,22) = 10.24, *p* =  0.0041) and egress out of (F(1,22) = 11.36, *p* =  0.0028) the turbinates, by Repeated Measures Two‐Way ANOVA, such that Aβ+ individuals had less influx and slower clearance across time.

**Conclusion:**

The findings suggest that the nasal pathway may serve as a viable route for CSF drainage in humans, with brain amyloid deposition impairing the drainage efficiency. These results provide novel insights into the relationship between brain amyloid pathology and the nasal clearance mechanism, warranting further investigation into the role of peripheral neurovascular pathways as potential biomarkers for AD. Understanding these mechanisms could pave the way for developing innovative interventions aimed at enhancing drainage pathways and mitigating the impact of amyloid burden on neurodegenerative processes.